# Evaluation of neurotoxicity of drugs from morphological and electrophysiological endpoints using human iPSC-derived neural cell models

**DOI:** 10.3389/fcimb.2026.1728941

**Published:** 2026-02-11

**Authors:** Zhe Qu, Shuangxing Li, Jingru Qiu, Guitao Huo, YuLin Liu, Di Zhang, Yanwei Yang, Xingchao Geng, Zhi Lin

**Affiliations:** 1 Key Laboratory of Quality Control and Non-clinical Research and Evaluation for Cellular and Gene Therapy Medicinal Products, Institute for Safety Evaluation, National Institutes for Food and Drug Control, Beijing, China; 2 Institute for Biological Product Control, National Institutes for Food and Drug Control, Beijing, China

**Keywords:** electrophysiological activity, high-content imaging, hiPSC-derived neural cells, microelectrode array detection, neurite outgrowth, neurotoxicity

## Abstract

**Introduction:**

Genome-edited human induced pluripotent stem cells (iPSCs) were first generated in 2007, and have been applied in pharmaceutical research, development, and clinical therapy as an *in vitro* platform for personalised phenotyping and drug discovery. iPSCs can differentiate into multiple types of neural cells, the *in vitro* hiPSC-derived neural model may also be an effective tool for early screening and evaluating potential neurotoxicants. Neurotoxicity is one of the limiting factors in the clinical application of many drugs. However, there is currently no set of standardized *in vitro* evaluation procedures and regulatory guidance documents for preclinical safety evaluation of new drugs, and traditional animal testing methods, including histopathological examination and behavioural testing, are inadequate for assessing the therapeutic effects and neurotoxicity of neurotherapeutic drugs.

**Methods:**

In this study, we developed a hiPSC-derived neural model that is suitable for testing neurospecific morphology and electrophysiology endpoints.

**Results:**

The research results showed that oxaliplatin and emodin at concentrations higher than 10 μg/mL, phenytoin sodium at concentrations exceeding 50 μg/mL, acrylamide and amantadine at concentrations exceeding 100 μg/mL, as well as aconitine and isoniazid (100 μg/mL) have a toxic effect on neurite outgrowth (p<0.05, p<0.01). No significant neurite outgrowth toxicity was observed in any of the dose groups of ethambutol. In MEA detection, phenytoin sodium and amantadine reduced the Number of Spikes, Mean Firing Rates, Number of Bursts, and Synchrony Index in a concentration-dependent manner. Phenytoin sodium, amantadine, and nano-Iron oxide all exhibited potent inhibitory effects on neuronal firing. Ethambutol exhibited a time-dependent and dose-dependent excitatory/inhibitory effect. The effect of isoniazid on neural electrical activity shifted from inhibition to excitation with the increase of administration dose and the extension of exposure time. This model enables a more comprehensive evaluation of neurotoxicity in neurotherapeutic drugs, nano-pharmaceuticals, and environmental organic compounds from the perspective of changes in neurite outgrowth and neural network functionality.

**Discussion:**

The method has specific test endpoints and high sensitivity, and can achieve high-throughput drug screening, which is expected to be applied to the safety risk assessment and scientific supervision of drugs with potential neurotoxicity, especially various types of neurotherapeutic drugs.

## 1 Introduction

Neurotoxicity is an important aspect of the non-clinical safety evaluation of drugs. Of the around 37, 000 human prescription drugs included in FDALabel, around 400 carry black box warnings for neurotoxicity and related issues (Walker et al., 2018). Early screening and pre-clinical safety evaluation for the neurotoxicity of drugs may prevent significant R&D investment in subsequent clinical trials and the withdrawal of a product from the market. Only the guidelines for *in vivo* neurotoxicity assessment have been approved by the Organization for Economic Co-operation and Development (OECD) and the International Council for Harmonisation for Pharmaceuticals for Human Use (ICH) (OECD, 1997; OECD, 2007; ICH, 2020). Neurobehavioral observation and histopathological examination are currently used for *in vivo* evaluation of neurotoxicity. However, the disadvantages of animal testing include subjective interpretation of test results, high costs for long-term tests, and the inability to explain and evaluate the neurotoxicity of a drug based on its toxic mechanism of action.

Since 2003, the European Centre for the Validation of Alternative Methods (ECVAM) has proposed to establish a reliable *in vitro* toxicological testing system and regulatory system (Hartung et al., 2003). The OECD is also committed to developing new approach methodologies (NAMs) of developmental neurotoxicity (DNT) research, including *in vitro* (omics, cell-based, tissue-based, etc.) assays, in silico models, and other computational approaches. In June 2023, the OECD published preliminary recommendations on the evaluation of *in vitro* test battery data for DNT (Development OfEC-oa, 2023). In 2025, the U.S. Food and Drug Administration announced that it would gradually phase out the animal testing requirements for monoclonal antibodies and other drugs (FDA, 2025). The *in vitro* cell culture models for high-throughput drug screening, organoids, organ-on-a-chip systems, and AI-based computational models of toxicity will undoubtedly be the future of drug regulatory science.

A variety of neuronal cell models, from neuronal or glial cell lines to primary neuronal and/or glial cultures, 3D cell culture models, and brain slices, from rodent cell models to human cell models, have been widely used to study the molecular and cellular mechanisms of Alzheimer’s disease (AD) (Essayan-Perez et al., 2019), seizures (Shirakawa and Suzuki, 2020), psychiatric disorders (Iwata, 2018; Nakazawa et al., 2019), and various neurological diseases or drug neurotoxicity evaluation. However, the *in vitro* neurotoxicity data obtained from different cell models are seldom included in the nonclinical safety evaluation reports submitted to drug regulatory authorities. Moreover, there are no guidelines for alternative *in vitro* methods to offer a validated assessment with specific, sensitive, and accurate model endpoints. Researching and validating various types of nerve cell models, especially human-derived cell models, to evaluate the safety of drugs with different neurotoxicity mechanisms of action, and conducting a comparative study with *in vivo* methods, can provide valuable data for the utilization of *in vitro* methods in the field of drug regulatory science.

The majority of the current data on nerve cell models comes from rodents. In order to address the limitations of extrapolating animal data to clinical situations and the ethical issues surrounding obtaining human-derived cells, several commercially available neural stem cells (NSCs) derived from human induced pluripotent stem cells (hiPSCs) may address the issue of species differences and cell resources. NSCs have the ability of self-renewal, which can be differentiated into three main neural cell types: neurons, astrocytes, and oligodendrocytes (Liu et al., 2023). NSCs have been utilized as a toxicological tool to evaluate developmental neurotoxicity. A study has shown that nickel inhibits the proliferation and differentiation of NSCs, which is associated with the up-regulation of p-c-Raf, p-MEK1/2, and p-Erk1/2 protein levels (Zhou et al., 2019). This study confirms the feasibility and reliability of stem cell-based systems for developmental neurotoxicity testing. NSCs differentiated in culture can serve as a model system for assessing the neurotoxicity of drugs. This can be achieved by examining cell viability, gene expression, mitochondrial aggregation, Neurite outgrowth and electrophysiological endpoints.

The differentiation time and growth density of stem cells, as well as culture media additives (such as growth factors, B27, N-2 Supplement, etc.), may affect the ratio of differentiated cells, promote Neurite outgrowth, and develop more mature electrophysiological functions. The differentiation of neural stem cells (NSCs) *in vitro* can mimic the *in vivo* development process of the nervous system and be utilized to assess neurotoxicity and developmental neurotoxicity. The morphological endpoints of the *in vitro* models are sensitive enough to detect direct damage to nerve cells and related neurological symptoms. For instance, the disruption of neurite outgrowth and synaptic connectivity in cultured neurons can indicate cognitive deficits (Huang and Song, 2019). The high-content screening platform combines the automation of microscopy image acquisition and analysis in a single system to quantify cellular events of interest (Lim, 2017). For example, it can quantify relevant parameters of neurite outgrowth, such as the number of neurites, length, and degree of branching. The bioelectrical activity of neuronal networks is very intense, and electrophysiological endpoints can be used to monitor the function of the nervous system. They can record the comprehensive response of various organelles to external stimuli in cells *in vitro*, including changes in mitochondria, antibody receptors, and ion channel activity. The microelectrode array (MEA) technology has been used to screen potential toxic compounds *in vitro* by recording the characteristics of the mean firing rate (MFR), bursting, synchrony, oscillations of network activity, and mutual information contained in the network (Ball et al., 2017). The following test substances were selected for this study: Oxaliplatin: a chemotherapy drug with well-documented neurotoxic side effects (Walker et al., 2018), oxaliplatin-induced peripheral neurotoxicity is a severe dose-limiting clinical problem that might lead to treatment interruption (Cheng et al., 2023); Aconitine: an intensely poisonous alkaloid, derives from many medicinal plants such as Aconitum carmichaelii Debx., Aconitum kusnezoffii Reichb, which were used to rheumatic fever, painful joints and some endocrinal disorders (Tang et al., 2018), and aconitine can cause neurotoxicity toxicity in both animal models and humans (Gao et al., 2022); Emodin: a natural anthraquinone derivative with diverse pharmacological activities (Wang, 2007); Isoniazid: an anti-tuberculosis drug that induces peripheral neurotoxicity owing to vitamin B6 deficiency (Badrinath et al., 2025); Ethambutol: a first-line antiobiotic for the treatment of tuberculosis and other Mycobacterium avium complex infections, optic neuropathy is a major adverse event of ethambutol treatment and may lead to irreversible visual loss (Cheng et al., 2025); Phenytoin sodium: a commonly used antiepileptic drug, has well-known neurological side effects of phenytoin therapy include cerebellar atrophy, cerebral atrophy, and brain stem atrophy (Arora et al., 2021); Amantadine, the only approved compound for reduction of involuntary movements, so called dyskinesia, in fluctuating orally levodopa treated patients, mainly utilized as an add-on therapy to mitigate levodopa-related dyskinesia (Marmol et al., 2021); Acrylamide: a commonly used industrial chemical that is known to be neurotoxic to mammals (Park et al., 2021), it is produced by the Maillard reaction between reducing sugars and free amino acids during food processing (Zhao M. et al., 2022); Iron oxide nanoparticles: can serve as drug carriers with favorable toxicological profiles, yet existing studies have demonstrated that these materials may induce certain neurotoxic symptoms (Silva et al., 2023; Tripathy et al., 2025). The aforementioned test substances, including anti-tumour drugs, neurotherapeutic drugs, nano-pharmaceuticals, environmental organic compounds, and monomer components of traditional Chinese medicines, exhibit considerable representativeness. In order to evaluate the morphological structure and functional effects of multiple types of neurotoxins on the differentiated cell models of hiPSCs-derived neural stem cells.

## 2 Materials and methods

### 2.1 Cell origin

Nerve cells derived from hiPSC (isolated from female skin fibroblasts): NeuroEasy^®^ human neural stem cells 1×10^6^(Beijing Cellapy Biotechnology Co., LTD, China).

### 2.2 Chemicals

NeuroEasy^®^ human neural stem cells (adherent culture) resuscitation inoculation medium (CA2311100), maintenance medium (CA2312100), digestive solution (CA2313050) and NeuroEasy^®^ human neuron differentiation basal medium (CA2308050-1) and additives (CA2308050-2), maintenance basal medium (CA2309250-1)and additives (CA2309250-2), neural stem cell digestive solution (CA2304050), neuron differentiation working solution (CA2310009) were obtained from Beijing Cellapy Biotechnology. Mouse anti-β III Tubulin antibody (ab78078) was obtained from Abcam. Mouse anti-MAP2 (2a + 2b) (M1406) was obtained from Sigma-Aldrich. Goat anti-Mouse IgG (H+L) Highly Cross-Adsorbed Secondary Antibody, Alexa Fluor Plus 488 (UD277967), ProLong™ Gold Antifade Mountant with DAPI (P36941), and penicillin-streptomycin (2108964) were obtained from Thermo Fisher. Paraformaldehyde (0708A20), Triton X-100 solution (1%, sterile) (1213A19), and Poly-L-Lysine solution (0114A20)were obtained from Beijing Leagene Biotech. Fibronectin human plasma (SLBZ5857) was obtained from Sigma Aldrich. DMSO(RNBD9351) was obtained from Sigma. Goat serum (011520200430) was obtained from Beyotime Biotechnology. Universal total RNA extraction kit (HS0402), First Strand cDNA Synthesis Kit (HS0611), Real SYBR Mixture (HS0613), 2×Taq PCR Mastermix (HS0602), D2000 DNA Marker (HS0713) were obtained from Beijing Hooseen Biotechnology. Agarose (111860) was obtained from Spain Biowest. 10, 000×DuRed nucleic acid dye (D009-500µL) was obtained from Beijing Fluorescence Biotechnology.

The details of the test articles to be evaluated for neurotoxicity are listed in **Table 1**, involving anti-tumour drugs, neurotherapeutic drugs, nano-pharmaceuticals, environmental organic compounds, and two monomer components of traditional Chinese medicines. All test articles were resuspend with the neuron medium.

**Table 1:** Drugs/compounds information for evaluating neurotoxicity.

Drugs/compounds	Category/indication	PSN/Lot No.	Supplier
Oxaliplatin	anti-tumor drug	White powder, 20 mg/bottle Lot No. 100367-201903	NIFDC^*^
Emodin	antitumor, antimicrobial, immunosuppressive,	Yellow powder, 20 mg/bottle Lot No. 110756-201913	NIFDC^*^
Aconitine	analgesic, antiphlogistic, anaesthetic, hypotensor	White powder, 20 mg/bottle Lot No. 100348-201908	NIFDC^*^
Isoniazid	anti-tuberculosis/antibacterial/anti-depressant	White powder, 100 mg/bottle Lot No. 100439-201809	NIFDC^*^
Ethambutol	anti-tuberculosis drugs	White powder, 50 mg/bottle Lot No. 130675-201601	NIFDC^*^
Phenytoin Sodium	antiparkinsonian agent, antineuralgic agent	White powder, 100 mg/bottle Lot No. 100584-201804	NIFDC^*^
Amantadine	antiparkinsonian agent, antiviral agent	powder, 50 mg/bottle Lot No. 100426-201703	NIFDC^*^
Acrylamide	environmental organic compounds	White powder, 1 mg/mL Lot No. 20150427	Sinopharm Chemical Reagent Co., Ltd.
Iron oxide(II, III)	magnetic nanoparticles solution	5 nm avg. part. size (TEM), amine functionalized, 1mg/mL Fe in H_2_O, dispersion	Sigma

*standard reference material from NIFDC (National Institutes for Food and Drug Control).

### 2.3 Cell culture

#### 2.3.1 Neural stem cell thawing and passaging

The cells were thawed quickly and fully in aseptic and 37°C water bath, slowly added 4mL NeuroEasy^®^ human neural stem cells (adherent culture) resuscitation inoculation medium, and mixed, then centrifuged at 1000 rpm for 5 min. Discard the supernatant and add an appropriate amount of neural stem cells resuscitation inoculation medium again, resuspended and inoculated into the prepared culture flask. The neural stem cells at a concentration of 5×10^5^ cells/mL were evenly adhered to the wall after culturing for 24 h in a 37°C 5% CO_2_ incubator. Refreshed the appropriate amount of human neural stem cell maintenance medium twice a week. The cells were continuously passaged for three generations when cell growth confluence reached 100%.

#### 2.3.2 NSCs differentiation into neural cells

The fibronectin-coated dishes/plates were incubated for at least 2 h at 37°C and 5% CO_2_. NSCs were incubated in digestive solution at 37°C for 5 min, and then the digestion was terminated in the neuron differentiation basal medium. The neural stem cells at a concentration of 1×10^5^ cells/mL were evenly adhered to the wall and cultured. After 24 h, the supernatant was discarded and replaced with a neuron differentiation maintenance medium to continue the culture, which was refreshed twice a week.

### 2.4 Characterization of hiPSC-derived neural cells

#### 2.4.1 Immunocytochemistry and high-content imaging

The staining and imaging of neural cells were conducted on the 7th, 14th, 14th to 21st, and 21st day after the day when the stem cells were initiated into the differentiation project. The neuronal derivatives were fixed with cold 4% paraformaldehyde for 20 min, and permeabilized in 0.2% triton-X-100 buffer for 15 min at room temperature. The cells were gently washed in 1× PBS, and then incubated in 5% goat serum blocking buffer at 37°C for 30 min to prevent the nonspecific binding of antibodies. The blocking buffer was removed, and the cells were incubated overnight at 4°C with mouse anti-βIII tubulin antibody(final dilution 1:500), mouse anti-MAP2 antibody(final dilution 1:500)in blocking buffer. The cells were washed 3 times with 1× PBS and incubated at 37°C for 1 h in blocking buffer containing fluorochrome-conjugated goat anti-mouse Alexa Fluor Plus 488. The nuclei were counterstained with 1 μg/mL DAPI dye for 5 min. The cells were washed 3 times and placed in the high-content imaging system for image capturing.

#### 2.4.2 Quantitative real-time PCR analyses

Neural cells were separately harvested on the 7th, 14th, 21st, and 28th day after the day when the stem cells were initiated into the differentiation project. The expression levels of specific protein markers of neural stem cells, neurons, and astrocytes, as listed in **Table 2**, were detected at four time points of differentiation of neural stem cells into neural cells to identify the developmental degree of neuronal culture. According to the manufacturer’s instructions, the total RNA was isolated using the universal total RNA extraction kit, and the concentration and purity of the isolated RNA were measured using a Nano-100 microspectrophotometer. The 200 ng of total RNA were reverse transcribed into cDNA each time using the first-strand cDNA synthesis kit. Quantitative real-time PCR (Q-PCR) was performed with SYBR Green technology. The β-actin was normalized as a reference gene and used undifferentiated NSCs for the calibrating conditions (ΔΔCt method).

**Table 2:** Markers of neurons and astrocytes differentiated from neural stem cells detected by Q-PCR.

Cell type	Marker/antibody	Description
Neural Stem Cells	nestin	Intermediate filament protein specifically expressed in neuroepithelial stem cells
Neurons	β-tubulinIII (Tuj-1)	The earliest neuronal skeletal protein expressed in the neuroepithelium
	synaptophysin	Neuronal protein located in synapses; indicates connections between neurons
Astrocytes	vimentin	Intermediate filament protein present in naive astrocytes
	GFAP	Glial fibrillary acidic protein as a marker of astrocyte activation
	Glutamate transporter	Transport chemicals such as glutamate across cell membranes while allowing water and chloride ions to pass through ^[12]^, representing the maturation of neuronal and astrocyte transport functions

#### 2.4.3 MEA detection

Neural field potential recordings were performed using the Maestro Pro MEA platform (Axion BioSystems, USA) at 37°C and 5% CO_2_. Real-time data was collected simultaneously across all 768 electrodes (12.5 kHz sampling rate, 200–3000 Hz band-pass). Analysis was performed using AxIS Navigator software 1.5 (Axion BioSystems) and an adaptive spike detection threshold set at 6 times the standard deviation of the background noise for each electrode with 1 second binning. Before detection, ensure that the temperature in the environmental chamber stabilises at 37°C and that the CO_2_ concentration stabilises at 5%. Ideally, the test should start when stable discharge commences after the cells have been placed in the instrument (in the operating procedure, we set this time to 10 minutes after the cells have been placed in the environmental chamber). Record for five minutes without using automatic stimulation. Additionally, to avoid the impact of medium replacement on neuronal activity measurements, do not change the cell culture medium within 24 hours prior to testing. The algorithm used for the network burst detection was ‘Inter-Spike Interval (ISI) Threshold’, an electrode burst is defined as at least 5 spikes on an electrode, each separated by an inter-spike interval (ISI) of no more than 0.1 seconds.

**Figure 1:**
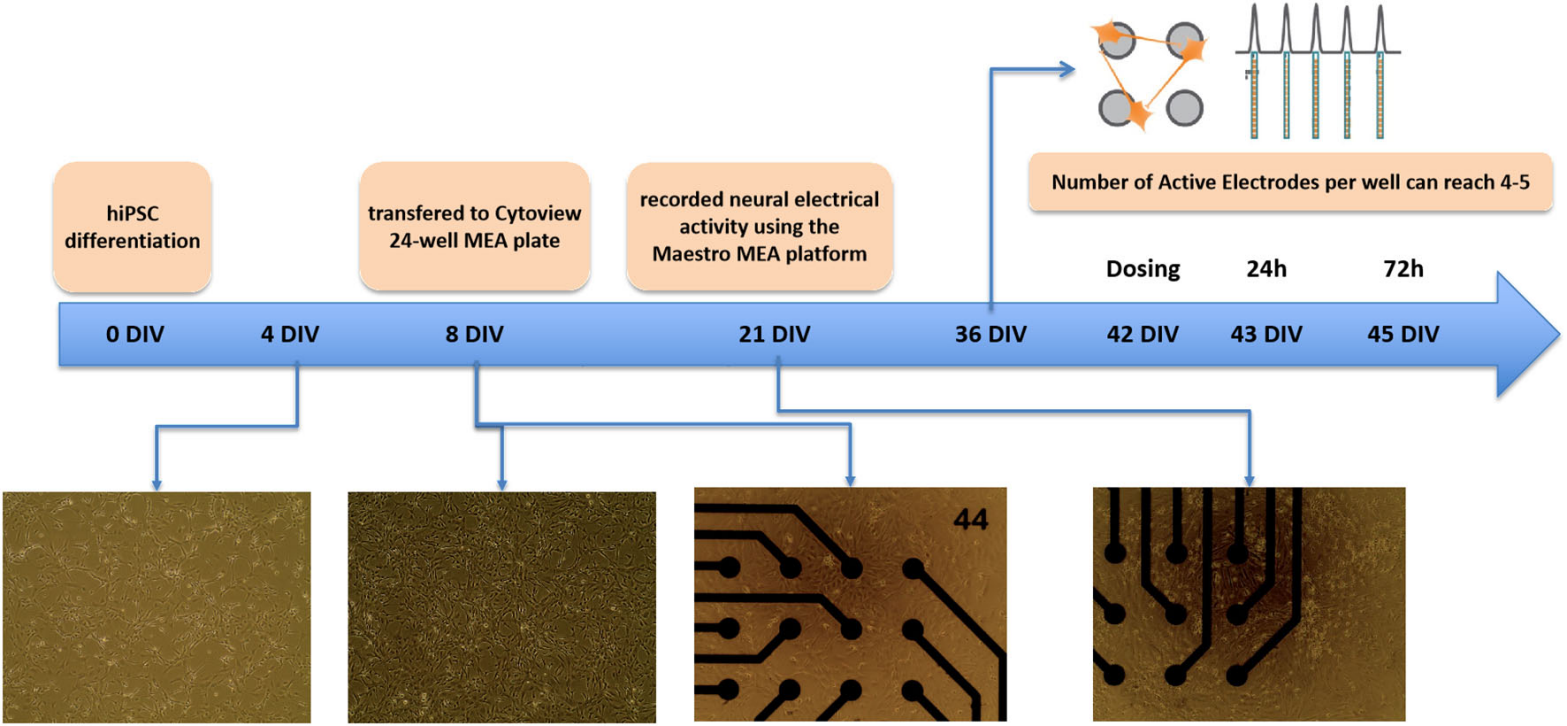
Neural network and neuronal firing formation. The neurons, day 8-differentiated from NSCs, were transferred to a cytoview 48-well MEA plate. A neural spike was detected from the 21st day of cell differentiation until the 42nd day of neuron culture. At this time, about four active electrodes each appeared in most wells of the 24-well plate.

When the proliferation of neural cells significantly decreased, the nerve cells (approximately 1×10^5^ cells/well) were transferred into Cytoview 24-well MEA plates at day 8 *in vitro* (DIV 8). Each MEA well within the 24-well plate contained 16 extracellular recording electrodes and a ground electrode. The neurites gradually elongated within 13 days after cell transfer. Neuronal action potentials/spikes, bursts, synchrony index, and active electrodes were recorded using the Maestro Pro and Maestro Edge recording systems (Axion Biosystems). At 21 days after cell differentiation, electrophysiological activities were measured on the Maestro every three days, and the detection continued until the administration on day 42 (DIV 42) (as shown in **Figure 1**).

### 2.5 Drug neurotoxicity evaluation

#### 2.5.1 High content imaging and neurite outgrowth assay

**Figure 2:**
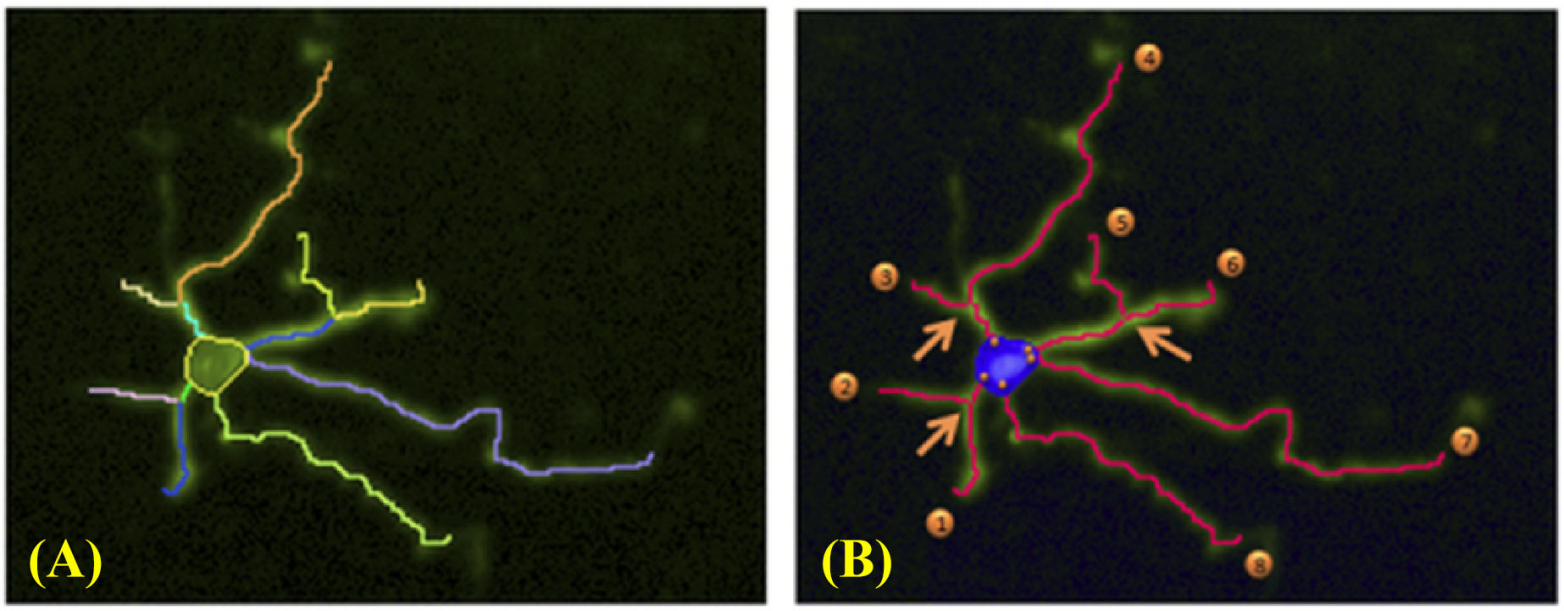
Sample images of neurite outgrowth analysis parameters. **(A)** Neurites captured by Harmony workflow. Number of Segments: number of neurites captured by Harmony with different colors; **(B)** Explanations of neurite outgrowth analysis parameters: Maximum Neurite Length: the longest neurite on the soma; Total Neurite Length: the total length of all neurites on the soma; Number of Extremities: as marked by the numbered orange circles; Number of Roots: as marked by the orange circles inside the soma; Number of Nodes: as marked by the arrows.

The neurite outgrowth assay was based on the use of the Operetta CLS high content platform in conjunction with Harmony workflow software to capture neuronal profiling. Six neurite outgrowth analysis parameters were showed in **Figure 2**. We performed the experiment using a 96-well plate, with 4 replicate wells set up for each group. Nine images were captured/well, and a total of 36 images/group were analyzed (9 images/well × 4 wells/group).

Neurite outgrowth in neural cells in 96-well plates after 48 h of drug treatment was immunostained with mouse anti-β-tubulin III antibody and corresponding secondary goat anti-mouse IgG using the immunocytochemical procedure described above 2.4.1. The cells used for the detection were mature neurons differentiated from neural stem cells over 21 days. The positive expression rate of anti-β III Tubulin was 98.99% ± 0.54% (95% confidence interval: 97.64%-100.3%), and the cell confluence reached 80%-90%. Six neurite outgrowth phenotypes including maximum neurite length, total neurite length, number of extremities, number of roots, number of segments, number of nodes were used to evaluate the effect of the nine drugs oxaliplatin, phenytoin, emodin, aconitine, isoniazid, ethambutol, amantadine, acrylamide, nano-iron oxide particles (II, III) on neurite outgrowth at a single administration of four dose levels listed in **Table 3** and four replicates of each dose level were performed.

**Table 3:** Four dose groups of each test article were set in the neurite outgrowth assay.

Test article	Blank control	Dose group 1	Dose group 2	Dose group 3	Dose group 4
Oxaliplatin	0 μg/mL	10 μg/mL	25 μg/mL	50 μg/mL	100 μg/mL
Emodin	0 μg/mL	10 μg/mL	25 μg/mL	50 μg/mL	100 μg/mL
Aconitine	0 μg/mL	10 μg/mL	25 μg/mL	50 μg/mL	100 μg/mL
Isoniazid	0 μg/mL	10 μg/mL	25 μg/mL	50 μg/mL	100 μg/mL
Ethambutol	0 μg/mL	10 μg/mL	25 μg/mL	50 μg/mL	100 μg/mL
Phenytoin Sodium	0 μg/mL	10 μg/mL	50 μg/mL	100 μg/mL	200 μg/mL
Amantadine	0 μg/mL	10 μg/mL	50 μg/mL	100 μg/mL	200 μg/mL
Acrylamide	0 μg/mL	10 μg/mL	50 μg/mL	100 μg/mL	200 μg/mL
Iron Oxide(II, III)	0 μg/mL	10 μg/mL	50 μg/mL	100 μg/mL	200 μg/mL

#### 2.5.2 Electrophysiological measurements

In this section, we selected Mean Firing Rate, Number of Bursts and Synchrony Index as the representative indicators to reflect the changes in neuronal electrical activity.

Differentiated neural cells were cultured in three Cytoview 24-well MEA plates for 42 days until neurons in the majority of wells developed stable synchronous firing. At this time, amantadine, ethambutol, isoniazid, phenytoin sodium, and iron oxide (II, III) were administered to evaluate how the drugs altered the electrophysiological activity of the neuronal network. The doses of amantadine, ethambutol, and iron oxide (II, III) were 10 μg/mL, 25 μg/mL, and 50 μg/mL; the doses of isoniazid were 2 μg/mL, 10 μg/mL, and 25 μg/mL. The doses of phenytoin sodium were 10 μg/mL, 50 μg/mL, and 100 μg/mL. The electrophysiological activity of the neuronal network was detected at 24h and 72h after administration according to the above compound concentrations. Mean Firing Rate, Number of Bursts, and Synchrony Index were recorded to evaluate the effect of the five drugs: amantadine, ethambutol, isoniazid, phenytoin sodium, and iron oxide (II, III) on neural electrophysiological activities at a single administration of three dose levels listed above. MEA detection follows the section 2.4.3.

### 2.6 Statistical analysis

Shapiro-Wilk was used to assess data normality, then data were analysed by one-way analysis of variance (ANOVA) using Bartlett’s test, followed by Dunnett’s test for multiple comparisons. The results are expressed as the mean ± SD. *p ≤* 0.05 was considered statistically significant. All the statistical analyses were performed with SPSS or GraphPad Prism 8 software. Four replicate wells were set for each group of the neurite outgrowth assay, and these analyses were from the same plates. In the MEA experiment, three replicate wells were set for each group. Considering that it is difficult to achieve simultaneous stable firing in multiple culture ells at the same time, some effective wells with above four active electrodes were used to detect and record the changes in electrophysiological indicators, ultimately, a single well was assigned to each dose group.

## 3 Results

### 3.1 Immunofluorescence identification of hiPSC-derived neural cell model

**Figure 3:**
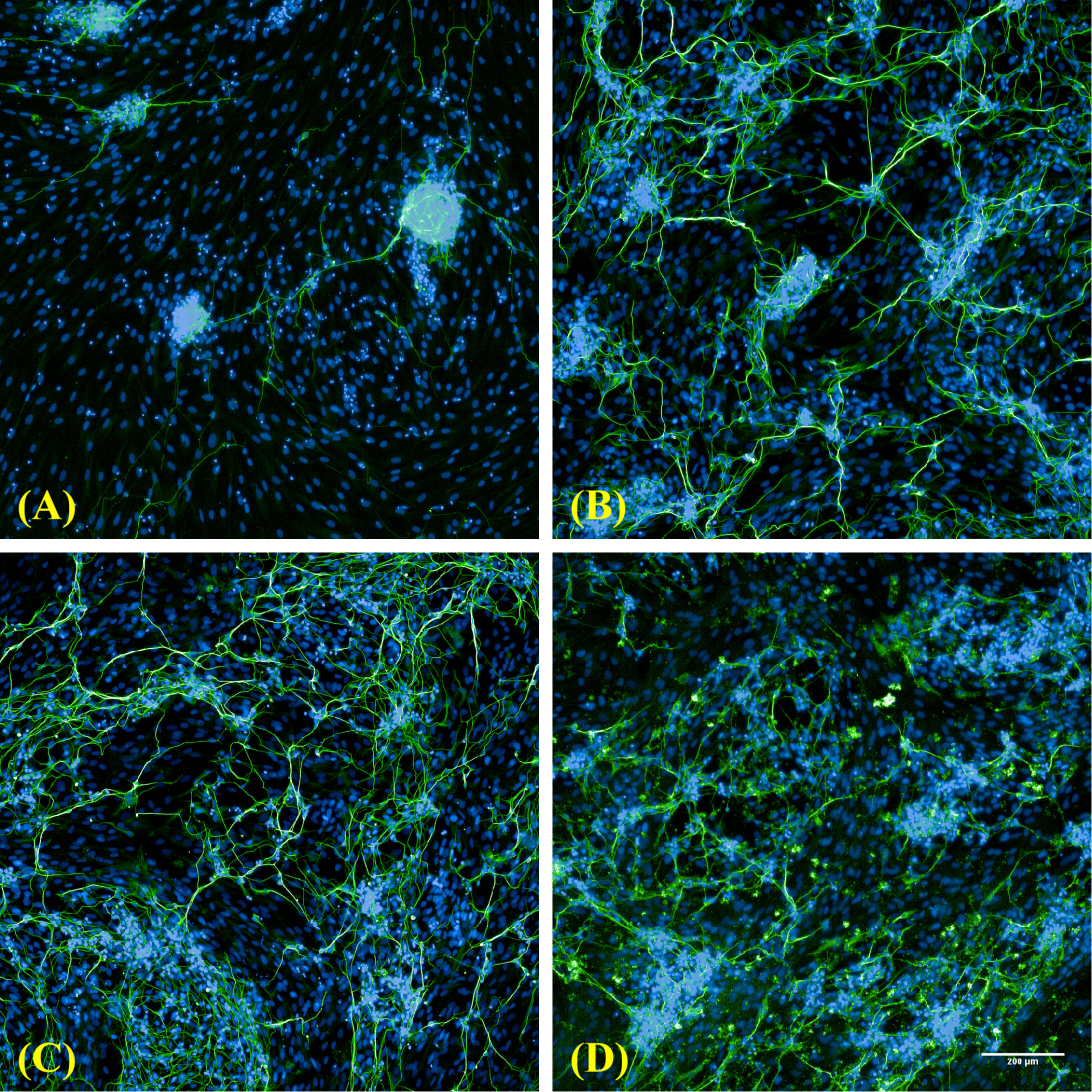
Morphological characterization of neurons day 7 to 21-differentiated from NSCs. **(A)** A few neurites have developed early at DIV 7. (200× magnification); **(B)** Neural networks were built through connections between neurons (synapses) at DIV 14. (200× magnification); **(C)** It showed that synaptic growth and maturation were accelerated during DIV 14 to 21. **(D)** The neuronal dendrites have begun to grow at DIV 21. (200× magnification). The β-tubulin III staining showed positive green fluorescence, which marks the synapses of neurons in the Figure **(A–C)**; The MAP2 staining showed positive green fluorescence, which marks the neuronal dendrites in the Figure **(D)**; The nuclei staining showed positive blue fluorescence.

NSCs were cultured/differentiated for up to DIV 21 and labelled with β-tubulin III and microtubule-associated protein 2 (MAP2) antibodies at DIV 7, 14, 21 to identify the morphology of neural network formation. A few neural cells began to develop small neurites at DIV 7. Many neurites extended to form dense neuropil and gradually differentiated into mature neurons between DIV 14 and DIV 21. The neurons were stained positive for the neuronal marker β-tubulin III and MAP2 (as a specific marker of neuronal dendrites) at DIV 7, 14, and 21(as shown in **Figures 3A-D**). MAP2 was expressed slightly later than β-tubulin III during neuronal development. Neurite outgrowth showed a significant increase by calculating the number of maximum neurite length, the number of total neurite length, the number of segments, the number of extremities, the number of roots, and the number of nodes from DIV 7 to DIV 21.

### 3.2 Neuronal differentiation analysed by neurospecific gene expression (Q-PCR)

**Figure 4:**
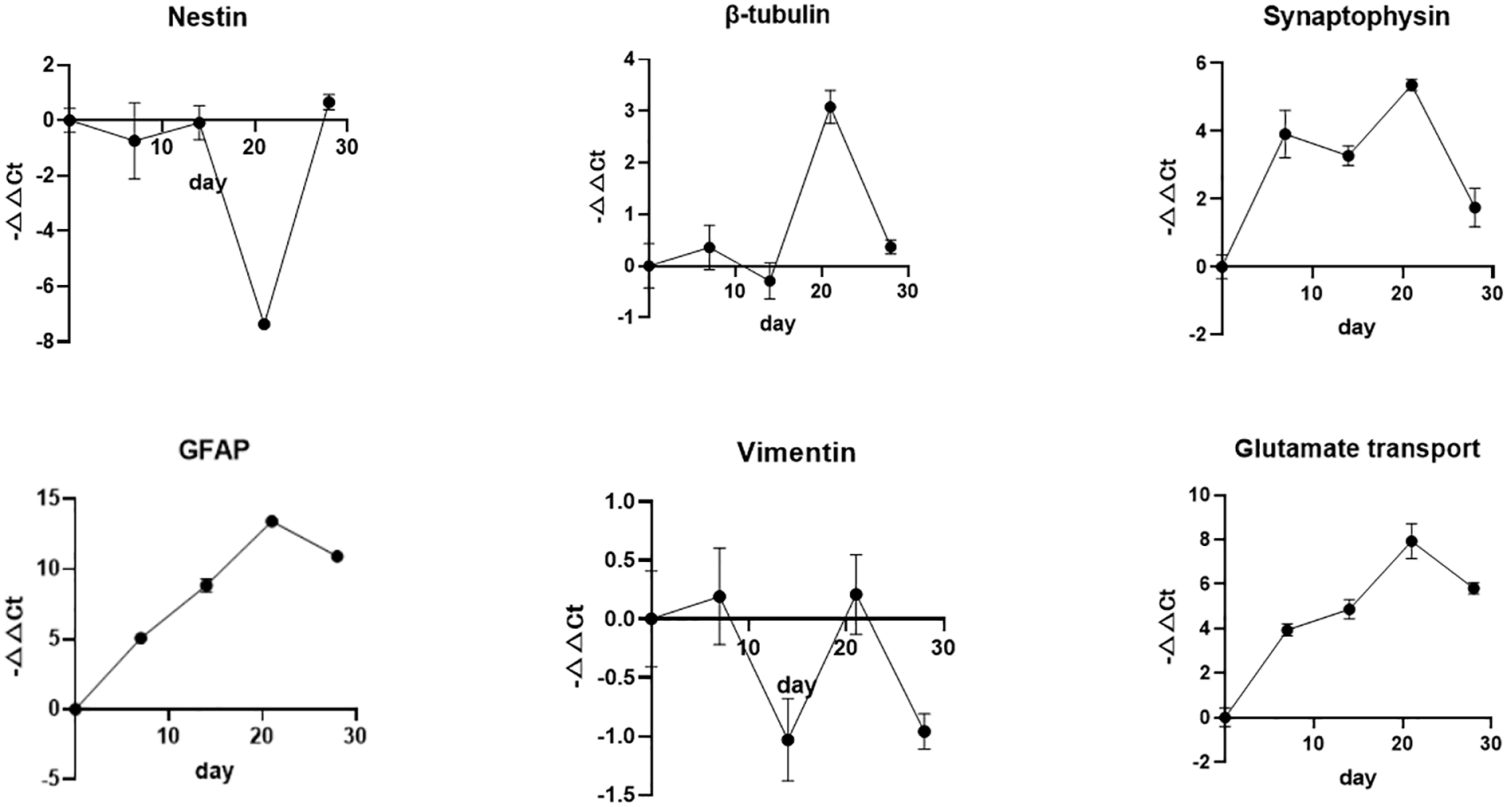
Characterization of nestin, β-tubulin III, synaptophysin, GFAP, vimentin, glutamate transporter gene expression of neural cells from DIV 0 to 28. -ΔΔct>0 indicated that enhanced gene expression levels compared to the control group; -ΔΔct < 0 indicated that inhibited gene expression compared to the control group. x ± s, n=3).

There was no significant change in the mRNA expression of the neuronal markers nestin and β-tubulin III until DIV 14. The expression of the pre-synaptic vesicles marker synaptophysin increased gradually during DIV 7, but a slight decrease was observed between DIV 7 and DIV 14. A progressive increase in the mRNA expression of the glutamate transporter and glial fibrillary acidic protein (GFAP) was observed until DIV 14, and the expression of the immature astrocyte marker vimentin presented a tendency to decrease. At the earlier differentiation process, the proliferation of neural stem cells was relatively significant compared to the neuronal differentiation. Meanwhile, NSCs began to differentiate into astrocytes earlier than neurons. A significant increase in the mRNA expression of the β-tubulin III, synaptophysin and glutamate transporter was observed from DIV 14 to DIV 21 compared to the decrease in nestin expression, which suggested that NSCs had undergone extensive differentiation into neurons. The increasing tendency in the expression of the β-tubulinIII, synaptophysin, and the glutamate transporter was slowed down from DIV 21 to DIV 28, which demonstrated that the process of these protein syntheses had been almost completed and most neurons had already developed and matured at DIV 21. After this, an apoptosis increase may occur due to the multifocal aggregation of neural cells. The GFAP expression increased, accompanied by a decrease in vimentin expression, demonstrating that astrocytes had gradually developed and matured between DIV 14 and DIV 28 (as shown in **Figure 4**).

### 3.3 Electrical activity of neuronal networks monitored by MEA

**Figure 5:**
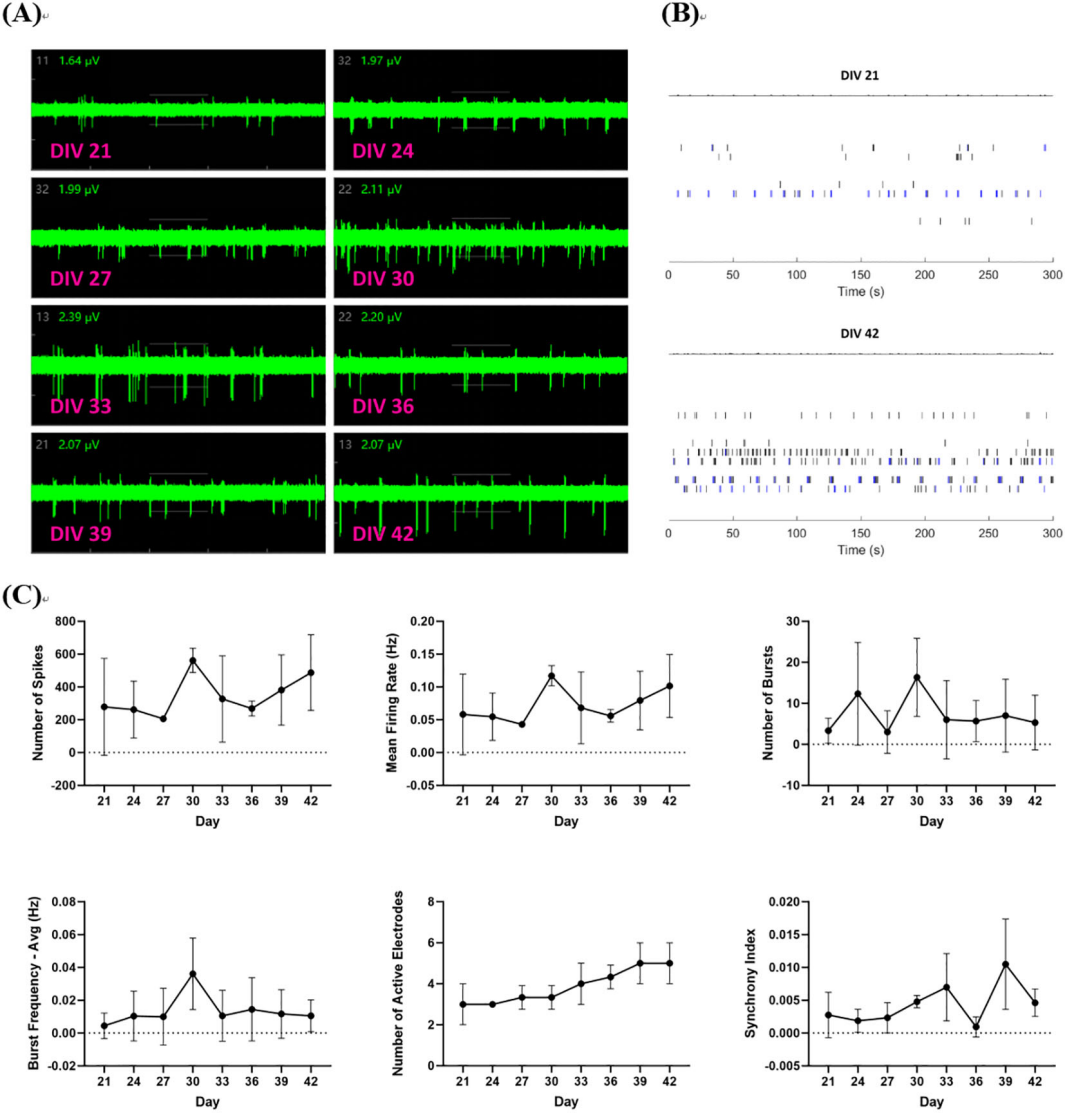
Waveform plots and parameters of spiking and bursting activity in neural networks. **(A)** Waveform diagram recorded by the MEA platform from DIV 21 to DIV 42; **(B)** Spike raster plots at DIV21 and DIV42 respectively were showed by depicting electrodes in a representative example well, with each tick representing one spike (field potential) in a 50 s interval; **(C)** Number of spikes, mean firing Rate, number of bursts, burst Frequency - Avg (Hz), number of active electrodes and synchrony index recorded by the MEA platform from DIV 21 to DIV 42. Waveform diagram and six parameters, number of spikes, mean firing Rate, number of bursts, burst Frequency - Avg (Hz), number of active electrodes and synchrony index from the MEA platform were recorded from unstable spikes of the neural networks that gradually exhibited synchronous bursting activity in the majority of 24 wells from DIV 21 to DIV 42. ( $\bar{x}$ ± s, n=3).

When the neuronal differentiation held a dominant position, the nerve cells were transferred to the Cytoview 24-well MEA plates on DIV 8. Thereafter, the cell culture was stabilised at 37°C in 5% CO_2_ atmosphere for 13 days and the neurites gradually extended, at which time the Maestro MEA platform was used to record neuronal electrophysiological activity. During DIV 21 to DIV 33, six parameters of firing, bursting, synchrony and active electrodes from MEA recordings showed an increasing trend, but the neuronal firing was relatively unstable and fluctuated significantly around DIV 30. Number of Spikes, Mean Firing Rate(Hz), Number of spikes and bursts, MFR and Burst Frequency-Avg (Hz) reached their peaks at DIV 30. From DIV 36, the spontaneous firing rate of nerve cells gradually stabilised over time, the neuronal action potential intensified, and the Number of Active Electrodes per well can reach 4-5, but the Synchrony Index showed certain fluctuations at a relatively low Index. Considering the spiking and bursting activity of most wells in the MEA 24-well plate and the number of active electrodes comprehensively, it was used for drug detection after 42 days of cell culture. (as shown in **Figure 5**).

### 3.4 Toxic effects of chemicals on neurite outgrowth

**Figure 6:**
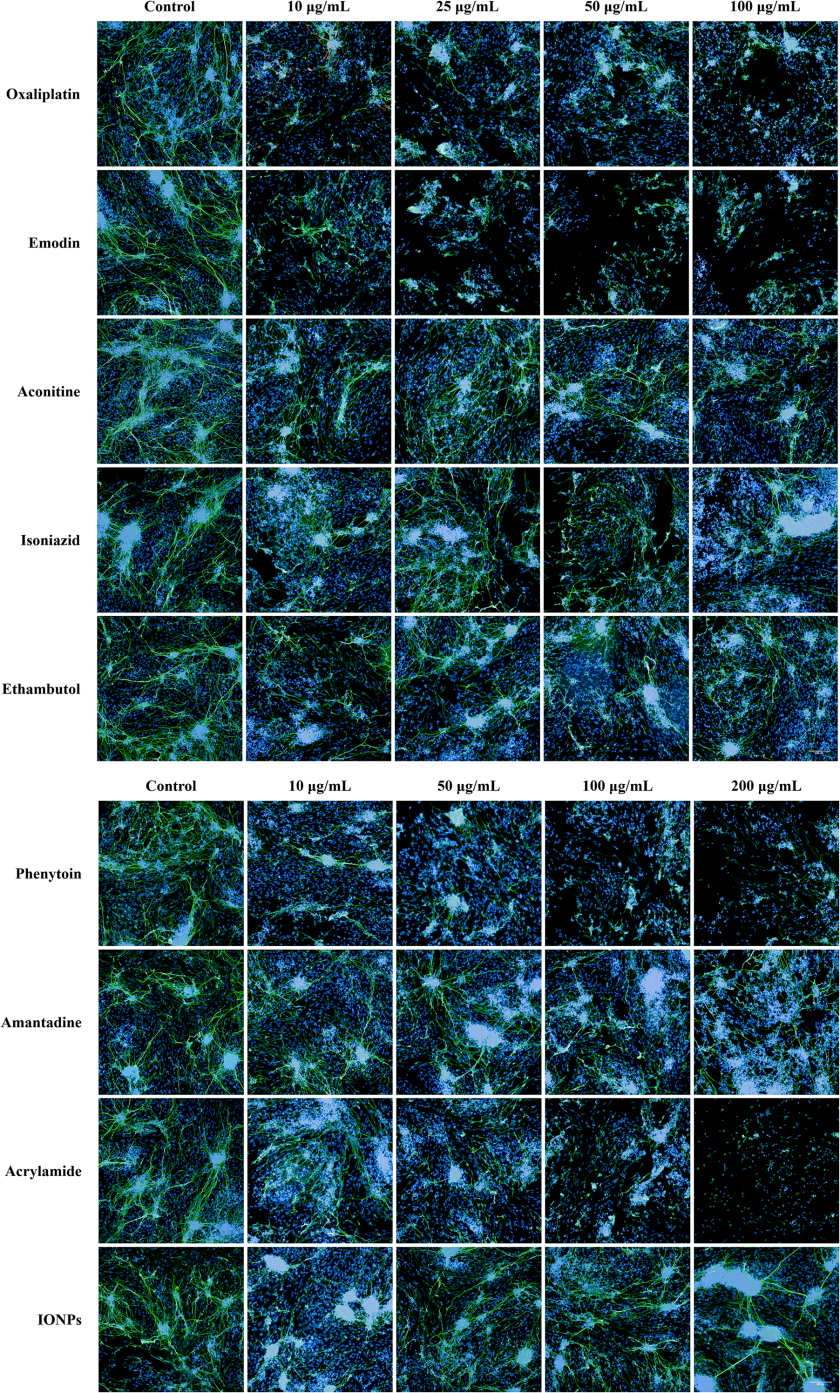
Fluorescence microscopy images of the dose-dependent toxic effects of oxaliplatin, emodin, aconitine, isoniazid, ethambutol, phenytoin sodium, amantadine, acrylamide, and iron-oxide nanoparticles after 48 h of exposure on neurite outgrowth. Images taken at 200× magnification, the neuronal dendrites labeled with β-tubulin III (green fluorescence) and nuclei labeled with DAPI (blue fluorescence).

**Figure 7:**
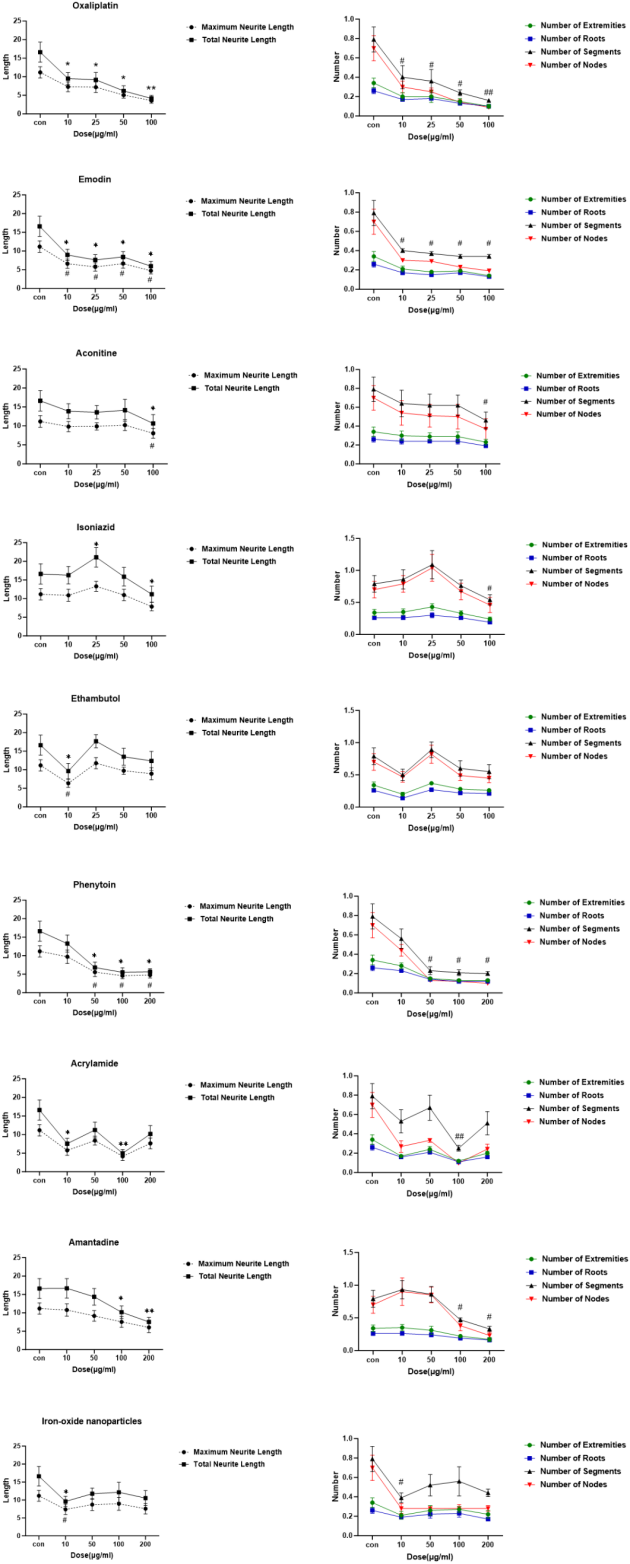
Six phenotypes of neurite outgrowth measured after 48 h of drug exposure. Data were analysed and compared to the control by one-way ANOVA (*/# = p < 0.05, **/## = p < 0.01) calculated from 10–200 mg/mL, including relative maximum neurite length, total neuritelength, number of extremities, number of roots, number of segments, and number of nodes. (x ± s, n=4).

Across three replicate experiments, exposure to oxaliplatin and emodin at concentrations higher than 10 μg/mL resulted in significant neurite damage, as well as more severe cell disintegration and necrosis. Aconitine (10 and 50 μg/mL) and isoniazid (10 and 50 μg/mL) did not have a toxic effect on Neurite outgrowth. However, at a concentration of 100 μg/mL, they were found to cause significant neurite damage (*p* < 0.05). No significant neurite outgrowth toxicity was observed in any of the dose groups of ethambutol. Phenytoin sodium and acrylamide, as well as amantadine at concentrations exceeding 50 μg/mL and 100 μg/mL, respectively, exhibited a significant toxic effect on Neurite outgrowth (p<0.05 and p<0.05, p<0.01) compared to the negative control group. No dose-dependent toxicity effect on neurite outgrowth was observed in the 10-200 μg/mL dose group of iron-oxide nanoparticles (IONPs). Chemical toxicity effects on Neurite outgrowth were evaluated by comparing measurements of maximum neurite length, total neurite length, number of extremities, number of roots, number of segments, and number of nodes with those of the negative control group. (as shown in **Figures 6**, **7**).

### 3.5 Electrophysiological effects of drugs on hiPSC-derived neural cell model

Three main parameters (Mean Firing Rate, Number of Bursts and Synchrony Index) from the MEA platform were used to quantitatively analyse the electrophysiological properties of neural networks treated with five chemical or nano-pharmacological test articles that disrupt nervous system activity. Mean Firing Rate (MFR) is a common metric in neuroscience to quantify the average activity of a neuron over a given period. It is calculated as: Mean Firing Rate=Number of Spikes/Duration of the analysis (Hz). Number of Bursts is the total number of single-electrode bursts over the duration of the analysis. For a well, the total number of electrode bursts across all electrodes in a well is reported. Synchrony Index is a unitless measure of synchrony between 0 and 1 (Paiva et al., 2010). And values closer to 1 indicate higher synchrony. Then, the Normalized Multiinformation (NMI) was used as a measure of complexity and synchronization in developing networks relative to several alternative methods, and compared to mean firing rates of neurons and synchrony index in this study (Ball et al., 2017). Firing, bursting, and synchrony are easily extractable from the data of MEA recordings, and also relatively sensitive metrics to evaluate neurotoxic actions of drugs and chemicals.

#### 3.5.1 Phenytoin sodium

**Figure 8:**
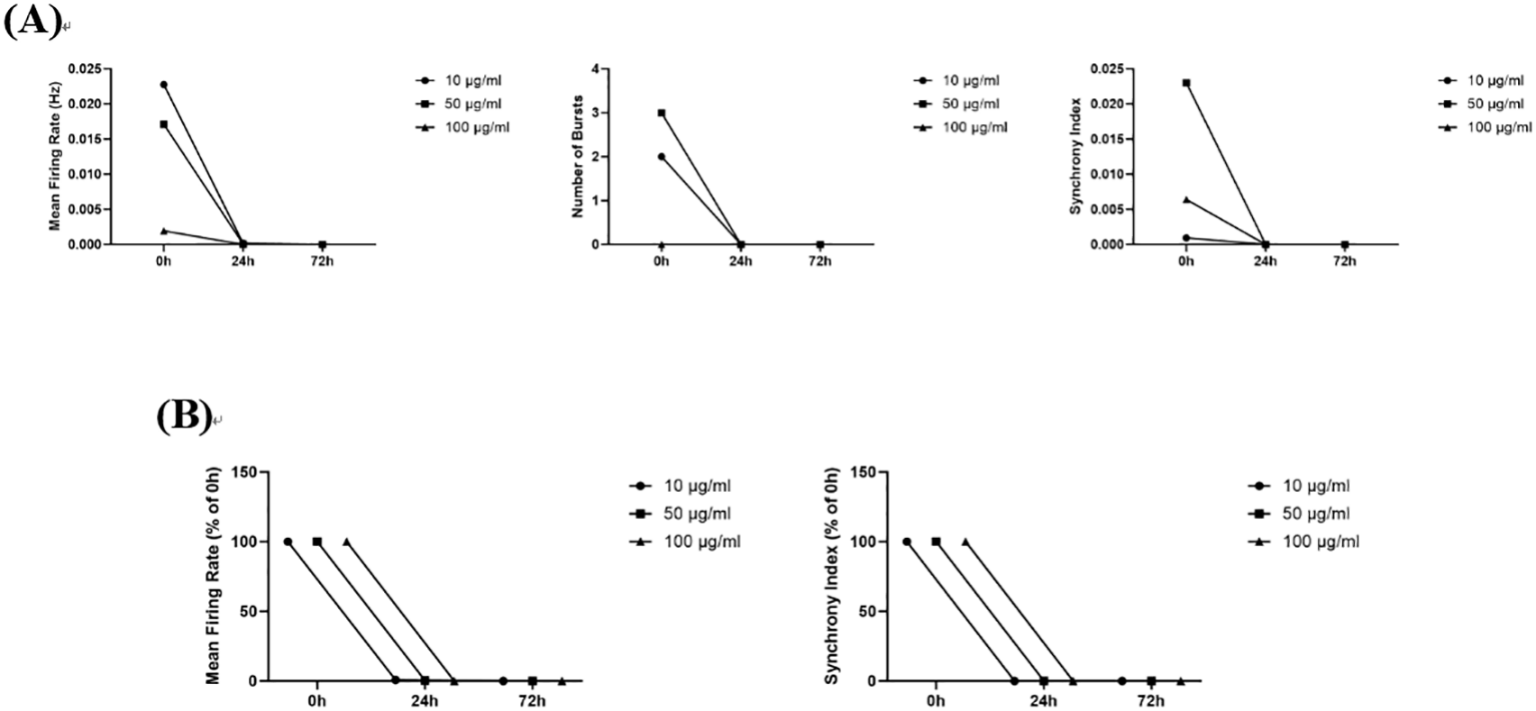
The effects of Phenytoin sodium on neuronal electrophysiology. **(A)** Changes in Number of Spikes, Mean Firing Rates, Number of Bursts, and Synchrony Index in neurons treated with 10 μg/mL, 50 μg/mL, and 100 μg/mL phenytoin Sodium for 0 h, 24 h, and 72 h; **(B)** Normalized results of Number of Spikes, Mean Firing Rates, and Synchrony Index.

In this study, compared to the 0 h administration time point, 10 μg/mL, 50 μg/mL, and 100 μg/mL phenytoin sodium reduced both the absolute and relative values of the Mean Firing Rates, Number of Bursts, and Synchrony Index at 24 h and 72 h post-administration, and all measured indices were reduced to zero in all dose groups at 72 h post-administration (**Figure 8**). Phenytoin sodium is a blocker of voltage-gated sodium channels, and its inhibitory effect on neuronal firing in this study may be the result of blocking sodium channels.

#### 3.5.2 Amantadine

**Figure 9:**
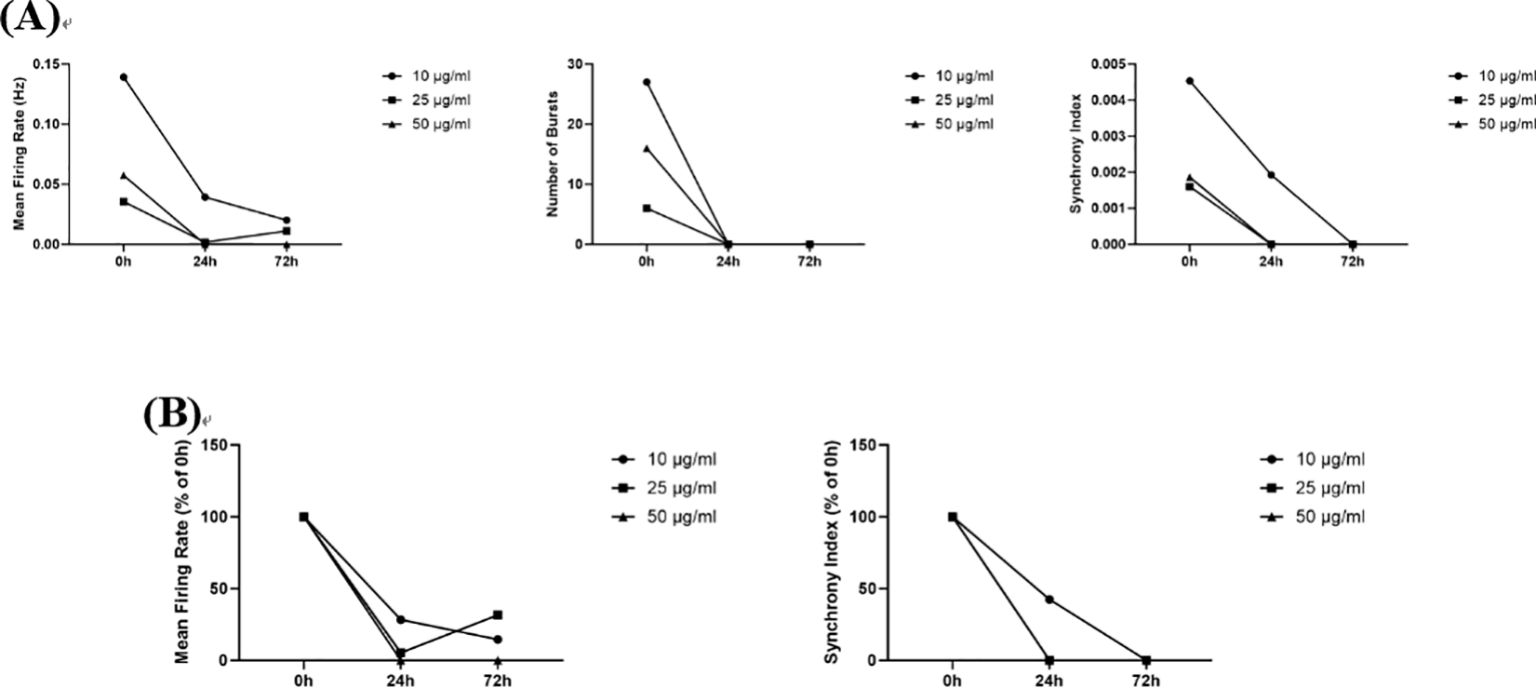
The effects of Amantadine on neuronal electrophysiology. **(A)** Changes in Number of Spikes, Mean Firing Rates, Number of Bursts, and Synchrony Index in Neurons Treated with 10 μg/mL, 50 μg/mL, and 100 μg/mL Amantadine for 0 h, 24 h, and 72 h; **(B)** Normalized Results of Number of Spikes, Mean Firing Rates, and Synchrony Index.

In this study, compared to the 0 h administration time point, 10 μg/mL, 25 μg/mL, and 50 μg/mL amantadine reduced the Number of Spikes, Mean Firing Rates, Number of Bursts, and Synchrony Index at both 24 h and 72 h post-administration. Although an increase in the Number of Spikes and Mean Firing Rates was observed at the 25 μg/mL dose at 72 h compared to the 24 h time point, an overall dose- and time-dependent inhibitory effect was evident (**Figure 9**). Current research indicates that amantadine acts as a non-selective antagonist of the N-methyl-D-aspartic acid (NMDA) subtype of glutamate receptors, thereby blocking glutamatergic neuronal activity (Blanpied et al., 2005). This mechanism may represent one of the underlying reasons for the inhibitory effect of amantadine on neuronal firing, as observed in the present study.

#### 3.5.3 Ethambutol

**Figure 10:**
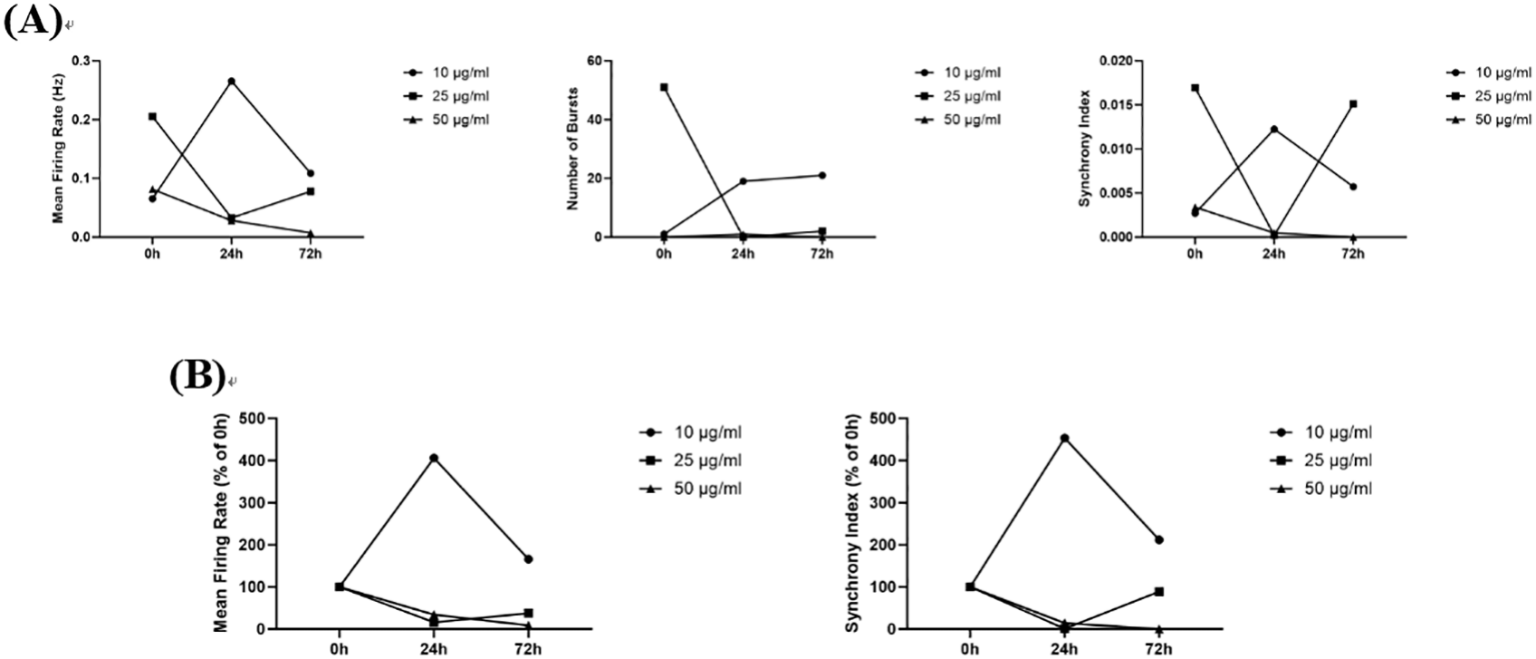
The effects of Ethambutol on neuronal electrophysiology. **(A)** Changes in Number of Spikes, Mean Firing Rates, Number of Bursts, and Synchrony Index in Neurons Treated with 10 μg/mL, 50 μg/mL, and 100 μg/mL ethambutol for 0 h, 24 h, and 72 h; **(B)** Normalized Results of Number of Spikes, Mean Firing Rates, and Synchrony Index.

Compared to the 0 h administration time point, 10 μg/mL ethambutol induced an increase in the Number of Spikes, Mean Firing Rates, Number of Bursts, and Synchrony Index at 24 h post-administration, and these values decreased at 72 h. In contrast, compared to 0 h, 25 μg/mL and 50 μg/mL ethambutol exhibited inhibitory effects on neuronal electrical activity at both 24 h and 72 h post-administration, which reduced the Number of Spikes, Mean Firing Rates, Number of Bursts, and Synchrony Index. It is worth noting that at the dosage of 25 μg/mL, all measured indices were elevated at 72 h compared to the 24 h post-administration (**Figure 10**). Ethambutol, a commonly used antitubercular drug, is clinically associated with optic neuropathy. This neurotoxic effect may occur via an excitotoxic mechanism, increasing the sensitivity of retinal ganglion cells to glutamate (Heng et al., 1999). In this study, ethambutol exhibited a time-dependent and dose-dependent excitatory/inhibitory effect, characterized by excitation followed by inhibition at low doses, inhibition followed by excitation at medium doses, and continuous inhibition at high doses.

#### 3.5.4 Isoniazid

**Figure 11:**
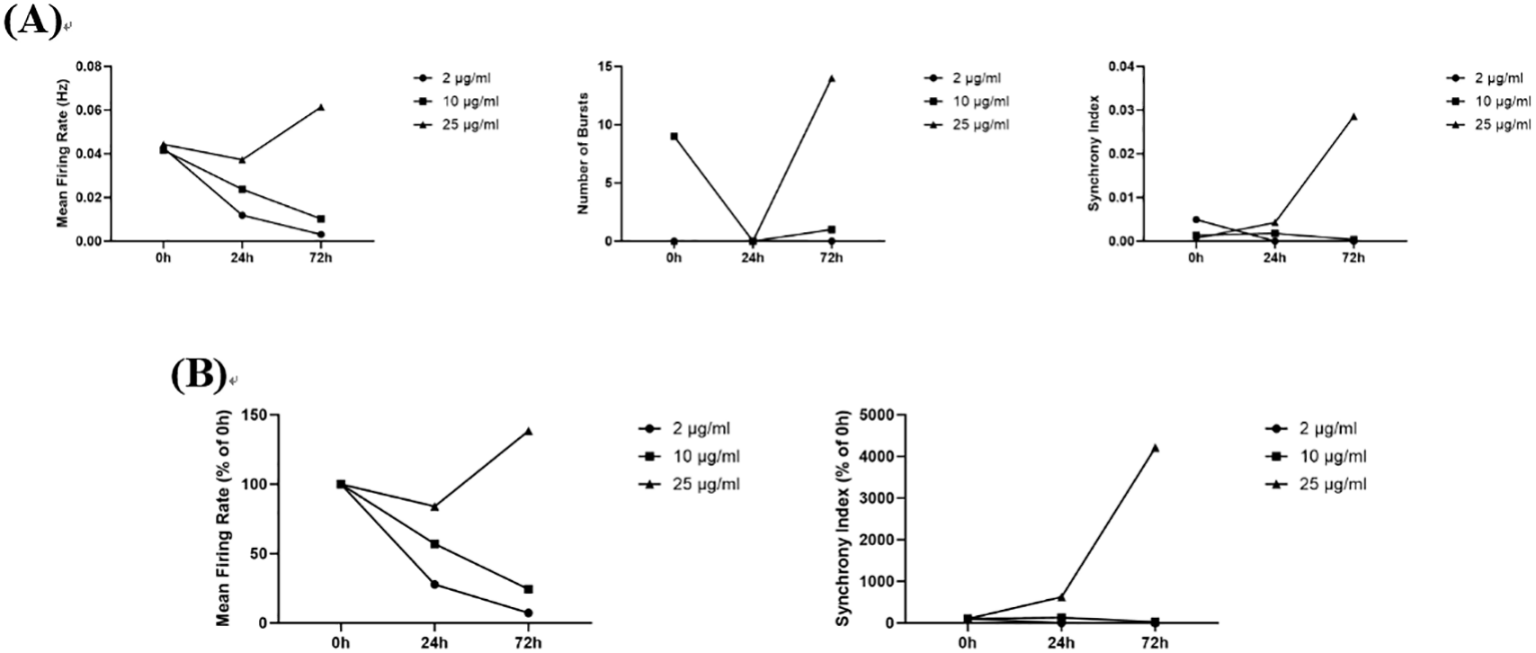
The effects of isoniazid on neuronal electrophysiology. **(A)** Changes in Number of Spikes, Mean Firing Rates, Number of Bursts, and Synchrony Index in Neurons Treated with 10 μg/mL, 50 μg/mL, and 100 μg/mL isoniazid for 0 h, 24 h, and 72 h; **(B)** Normalized Results of Number of Spikes, Mean Firing Rates, and Synchrony Index.

In this study, compared to the 0 h administration time point, 25 μg/mL isoniazid reduced electrophysiological parameter values at 24 h post-administration. However, with prolonged exposure, isoniazid exhibited a stimulatory effect on neuronal electrical activity at 72 h post-administration, increasing the Number of Spikes, Mean Firing Rates, Number of Bursts, and Synchrony Index. 2 μg/mL and 10 μg/mL isoniazid demonstrated time-dependent inhibitory effects on neuronal electrical activity, with the inhibitory trend being more pronounced at the 2 μg/mL concentration. Collectively, as both the administered dose increased and the exposure duration lengthened, the effect of isoniazid on neural electrical activity shifted from inhibition to stimulation (**Figure 11**). Isoniazid is a first-line antitubercular drug widely used clinically, with neurotoxicity being one of its principal adverse effects. Isoniazid can interfere with the synthesis of the inhibitory neurotransmitter gamma-aminobutyric acid (GABA) by inhibiting glutamate decarboxylase, inducing seizures and an increase in brain firing rate (Shukurova et al., 2022). The observed stimulatory effect on neural electrical activity with increasing drug exposure in the present study aligns with this established mechanism.

#### 3.5.5 Iron oxide(II, III)

**Figure 12:**
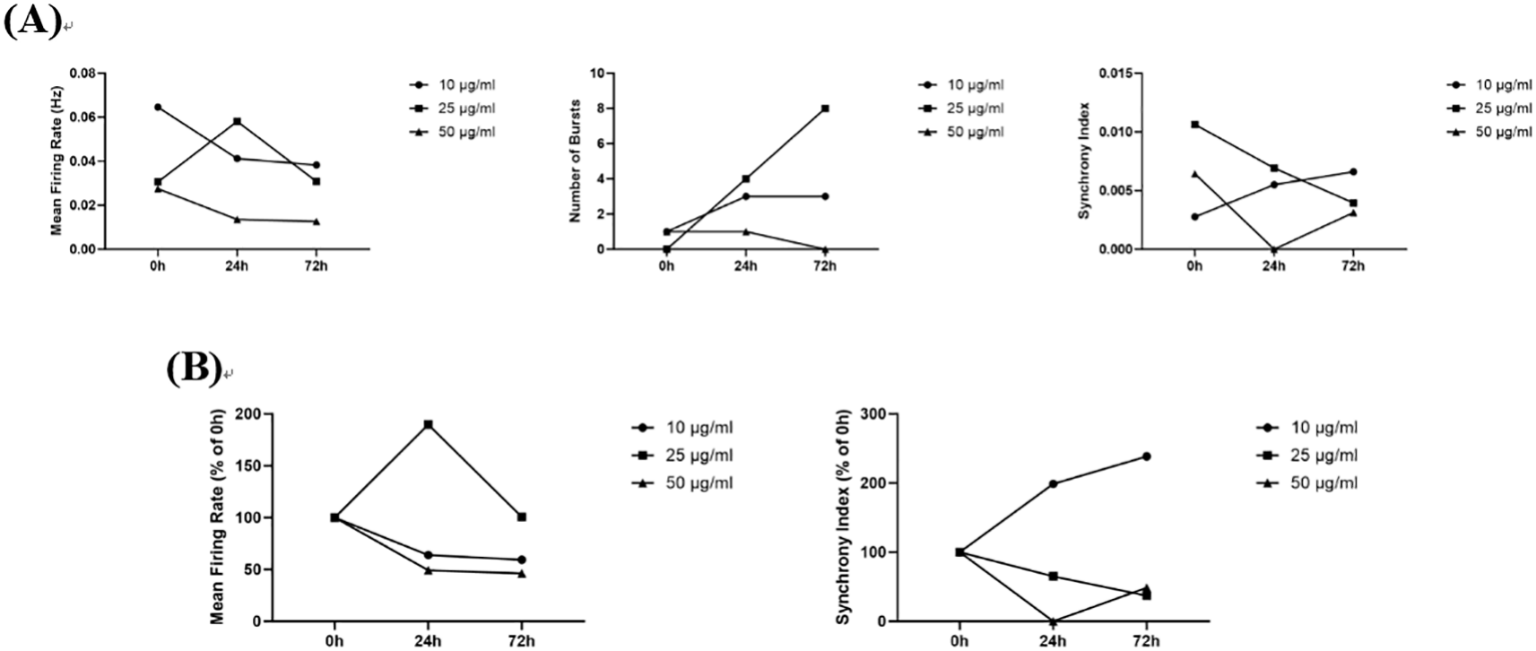
The effects of nano-iron oxide on neuronal electrophysiology. **(A)** Changes in Number of Spikes, Mean Firing Rates, Number of Bursts, and Synchrony Index in Neurons Treated with 10 μg/mL, 50 μg/mL, and 100 μg/mL nano-iron oxide for 0 h, 24 h, and 72 h; **(B)** Normalized Results of Number of Spikes, Mean Firing Rates, and Synchrony Index.

In this study, 10 μg/mL and 50 μg/mL nano-iron oxide exhibited time-dependent inhibitory effects on neuronal electrical activity, with the 50 μg/mL dose demonstrating more pronounced inhibition. At the 25 μg/mL dose level, however, the values for Number of Spikes, Mean Firing Rates, Number of Bursts, and Synchrony Index initially increased, followed by a decrease with prolonged exposure time (**Figure 12**). Badman (Badman et al., 2020) et al. demonstrated that dextran-coated nano-iron oxide at 20 μg/mL and 40 μg/mL concentrations markedly suppresses neuronal electrophysiological activity. The observed inhibitory effects of nano-IO on neuronal electrical activity at concentrations of 10 μg/mL and 50 μg/mL in the present study are consistent with previous findings. This suppression may be associated with reduced synaptic efficacy in neurons.

## 4 Discussion

The correlation between the proliferation and differentiation of neural stem cells will determine the developmental characteristics of the neural cell model. At present, there is no universally accepted model of human neural cell culture suitable for *in vitro* evaluation of the neurotoxicity of drugs. hiPSC-derived neural cells have functional synapses. On the one hand, the morphological changes of neurons can be evaluated by immunocytochemistry and high-content imaging to reflect the synaptic function; on the other hand, the functional activity of neurons can also be reflected by monitoring the electrophysiological activities. This study explored a hiPSC-derived neural cell culture model and a high-throughput toxicity screening system, which was a dual detection platform for evaluating both morphological and electrophysiological neurospecific indicators.

In this study, the expression dynamics of the astrocyte markers vimentin and GFAP were determined using neuron-specific gene expression profiling by ultrasensitive Q-PCR during neuronal differentiation. In the morphological assessment and inflammatory cytokine secretion assays conducted for the preliminary characterization of this model, the neuronal proportion reached 98.99% ± 0.54%, while the astrocyte proportion was below 10%, with no detectable pro-inflammatory cytokine secretion. Thus, this model was defined as a high-purity, directionally differentiated neuronal model. The minimal astrocyte population exerted no significant interference with neuron-specific detection indices, including neurite outgrowth and electrophysiological measurements. In subsequent studies, if the model can be further optimized to more faithfully recapitulate the physiological microenvironment of human brain tissue, dedicated detection endpoints for glial cells may be incorporated.

In the *in vitro* model system, 1×10^5^ cells/mL was determined as the suitable cell inoculation density for evaluating the two neurospecific indexes. When the stem cell culture reached the highest cell differentiation rate and the lowest proliferation rate at the 1×10^5^ cells/mL density on DIV 21, the cell model at this time could be used to measure the synaptic length in the neurite outgrowth assay. In electrophysiological measurements, the density (1×10^5^ cells/well) of cells inoculated in the MEA plate exhibited a more orderly cell growth pattern, which was expected to be a potential alternative system for animal testing for acute or long-term toxicity studies of drugs.

Human and non-human cell lines or primary cells for neurite outgrowth have been widely employed as the *in vitro* model for neural regeneration research (Terzi et al., 2014; Shibato et al., 2021). In the study, human iPSC-derived neurons as *in vitro* models of neurite outgrowth had been demonstrated to be effective in evaluating a variety of neurotoxic chemicals, drugs and substances (neurotoxic agents). For example, oxaliplatin and acrylamide induced significant neuroaxonal injury and even cell death. Growth of the synapses of nerve cells was significantly inhibited in the high dose of phenytoin sodium, amantadine, aconitine and isoniazid. Emodin also showed significant neurocytotoxicity, which was contrary to the neuroprotective effect of emodin reported in some literature (Leung et al., 2020), and the protective dose levels or No Observed Adverse Effect Level(NOAEL) of emodin *in vitro* models need to be further explored. Ethambutol and IONPs did not have significant inhibitory effects on neurite outgrowth.

Microelectrode array (MEA) technologies have recently attracted attention due to their ability to noninvasively, rapidly and continuously measure the electrophysiological activity in neural networks. Neural network development, which covers a series of upstream neurodevelopmental events such as neuronal differentiation and neuroaxonal outgrowth, synaptogenesis, and neuron-glial interaction, is a more “top” or “broadband” endpoint that can be used in high-throughput screen potential neurotoxic compounds that are not detectable by other endpoints. A combination of individual spikes, spikes in bursts, and bursts that occur individually and in synchrony is optimal for testing compounds that cause a variety of neural network perturbations (Bradley and Strock, 2019). In this study, we selected Mean Firing Rate, Number of Burst and Synchrony Index, which comprehensively and representatively reflect the alterations in neuronal electrophysiological activity induced by the test substances from three distinct perspectives: Activity measures, measures of spike train organization, and measures of cross-channel synchronization. We evaluated the electrophysiological function of phenytoin sodium, amantadine, ethambutol, isoniazid, and iron-oxide nanoparticles, which are used to treat neurological disorders and have been shown to disrupt neuronal firing at high clinical doses. The results of the MEA test are related to the mechanism of action of the above test substances.

Phenytoin sodium is one of the most widely used antiepileptic drugs, but long-term use may induce dose-dependent adverse effects such as cerebellar atrophy, cerebral atrophy, and brain stem atrophy (Arora et al., 2021). Phenytoin sodium can induce the upregulation of Cytochrome P450 enzymes (CYP), leading to increased CYP-mediated testosterone (TES) metabolism, which has a negative impact on hippocampal neurogenesis and neuronal survival, causing depression and cognitive impairment in patients (Zhang et al., 2023). The present study demonstrated the inhibitory effect of phenytoin sodium on neuronal firing, the underlying mechanisms may involve both blockade of neuronal surface sodium channels and the induction of elevated intracellular CYP levels by phenytoin sodium. Current research indicates that amantadine acts as a non-selective antagonist of the N-methyl-D-aspartate (NMDA) subtype of glutamate receptors, thereby blocking glutamatergic neuronal activity (Blanpied et al., 2005). In this study, we observed that amantadine exhibited a dose-dependent inhibitory effect on neuronal firing, consistent with its mechanism of inhibiting NMDA. Ethambutol-induced neuropathy may result from impaired mitochondrial function in nerves (Kozak et al., 1998). In this study, ethambutol demonstrated a transient excitatory effect on neuronal activity at a low dose (10 μg/mL) and short exposure duration (24 h). This may be attributable to an ethambutol-induced increase in neuronal sensitivity to glutamate under these specific conditions. Conversely, ethambutol exerted inhibitory effects on neuronal electrical activity at higher doses (25 μg/mL, 50 μg/mL) and longer exposure times (72 h). This suppression may arise from progressive drug exposure leading to impaired mitochondrial function in neurons. Isoniazid, an anti-tuberculosis drug, can cause seizures at high doses, resulting in excessive neuronal firing. The results of this study show that isoniazid inhibited neuronal firing at 2 μg/mL and 10 μg/mL. However, when the dosage was increased to 25 μg/mL and the administration time was increased to 72 h, the Mean Firing Rate was higher than in the control group, indicating an enhancing effect, which was consistent with its mechanism of inhibiting GABA synthesis. Nano-iron oxide is a widely utilized nanomaterial, with commercially available iron oxide nanoparticle products primarily indicated for cancer treatment and iron-deficiency anemia. Lu (Lu et al., 2022) et al. reported that ultrasmall superparamagnetic iron oxide nanoparticles significantly inhibit long-term potentiation (LTP) in mice, thereby reducing synaptic efficacy, which may be the underlying mechanism of their inhibition of neuronal firing.

In this study, the majority of subjects demonstrated consistent effects on neurite outgrowth and neuronal firing. For example, phenytoin sodium and amantadine inhibited both neurite outgrowth and neuronal electrophysiological activity. However, for some test substances, there is no obvious correlation between the results of these two indicators. For example, isoniazid at all dose levels first showed an inhibitory effect on neuronal firing and then an excitatory effect, but it only has a significant inhibitory effect on neurite outgrowth at high dose levels. Ethambutol first showed an excitatory effect and then an inhibitory effect, but no significant effect was observed on neurite outgrowth. Additionally, it is noteworthy that iron-oxide nanoparticles did not significantly affect neurite outgrowth on the human iPSC-derived neurons, but showed inhibition on neural network firing. Here, electrophysiological measurement is a more sensitive index than neurite outgrowth for the evaluation of iron-oxide nanoparticles is more sensitive to the evaluation of iron-oxide nanoparticles than neurite outgrowth, indicating that they could only cause neuronal function damage (Imam et al., 2015). Neurite outgrowth inhibition reflects neuronal developmental toxicity. Excessive excitement and abnormal discharge of neurons are potential changes caused by abnormal activities of brain neurons, reflecting brain dysfunction (Crunelli et al., 2023; Neves et al., 2023). They are two characteristic indicators of neurotoxicity and have no direct correlation in toxicity evaluation, but are related to the compounds’ neurotoxic mechanism of action. Therefore, there should also be corresponding evaluation indicators for the specific and sensitive endpoints used to detect the *in vitro* neurotoxicity of compounds with different mechanisms of action. The results of this study also provide an important reference for setting *in vitro* evaluation indicators for multiple types of neurotoxins.

This study broke through the limit of traditional *in vitro* cell experiments, which only measured the cell viability (Wei et al., 2021) and function, as well as more advancedly it provides a comprehensive evaluation model of morphology and neural network electrophysiological activity specific index for high-throughput screening of neurotoxicants. It was used to evaluate the neurotoxicant-induced cell qualitative alteration and the functional changes regarded as the amplification of the pharmacodynamic effects in the nervous system, which was of great value for evaluating the side-effects and toxicity of neurotherapeutic drugs. The model was designed to provide the dual indicators to analyse neurotoxicity, which has the advantage of consistent experimental conditions and reliable background data. In recent years, a variety of new technologies such as *in vitro* cell models (Wang et al., 2022; Park et al., 2024), organoids (Cao et al., 2023; Hu et al., 2025), organ-on-a-chip (OOAC) (Castiglione et al., 2022; Kawakita et al., 2022), and computer simulation are mostly in the process of maturation and validation (Karakuş and Kuzu, 2025), and are expected to screen and evaluate the pharmacological and toxic effects of neurotoxins in complex *in vitro* models that are closer to the physiological state of the human or AI prediction models (Zhao X. et al., 2022; Rudroff, 2024; Lee et al., 2025). Although this study used a single neuron cell model and couldn’t comprehensively assess the neurotoxicity of the test substance from all aspects, such as the complex interaction among different cells, neuroinflammatory responses, and the interaction between the nervous system and other tissues and organs (such as the “gut-brain axis”). However, the research method still has certain practical application value, offering a balance between scientific rigor and economic feasibility.

## Data Availability

The datasets presented in this study can be found in online repositories. The names of the repository/repositories and accession number(s) can be found in the article/supplementary material.
